# An unprecedented antioxidative isopimarane norditerpenoid from bivalve clam, *Paphia malabarica* with anti-cyclooxygenase and lipoxygenase potential

**DOI:** 10.1080/13880209.2017.1280061

**Published:** 2017-01-24

**Authors:** Minju Joy, Kajal Chakraborty

**Affiliations:** Central Marine Fisheries Research Institute, Cochin, India

**Keywords:** C19 isopimarane norditerpenoid, marine bivalve, antioxidant, anti-inflammatory, selectivity index

## Abstract

**Context:** The yellow-foot bivalve clam, *Paphia malabarica* Chemnitz (Veneridae) is distributed in the southwest coastal regions of India. The ethyl acetate-methanol extract of this species exhibited significant antioxidant and anti-inflammatory activities.

**Objectives:** To purify and characterize the bioactive compound from *P. malabarica* along with *in vitro* assays.

**Materials and methods:** The edible portion of *P. malabarica* was freeze dried (1.20 kg, yield 20.0%) and extracted with ethyl acetate and methanol (1:1 v/v, 500 mL ×3) by sonication (8 h). The antioxidant activity against DPPH/ABTS^+ ^and anti-inflammatory potential against cyclooxygenase-1,2 (COX-1, 2)/5-lipoxygenase (5-LOX) enzymes were carried out with varying concentrations (0.25–2.00 mg/mL) to determine the IC_50_ values. The crude extract was chromatographically fractionated and the fraction showing greater potential was further fractionated to yield the pure compound, which was characterized by extensive NMR, IR and mass spectroscopic analyses.

**Results and discussion:** The fractionation of crude extract of *P. malabarica* was followed by structural characterization of the new rearranged isopimarane derivative, 18 (4 → 14), 19 (4 → 8)-bis-abeo C_19_ norditerpenoid. The isopimarane derivative displayed comparable antioxidant activity with α-tocopherol (IC_50_ DPPH scavenging activity ∼0.6 mg/mL), whereas anti-inflammatory (anti-5-LOX) effect of the title compound was significantly greater (IC_50_ 0.75 mg/mL) than ibuprofen (IC_50_ 0.93 mg/mL). In addition, the greater selectivity index (anti-COX-1_IC50_/anti-COX-2_IC50_ 0.85) explained the lesser side effects of the isopimarane norditerpenoid than the nonsteroidal anti-inflammatory drug-based therapies.

**Conclusions:** The isopimarane derivative isolated from *P. malabrica* can be a natural substitute to commercial drugs in future.

## Introduction

The oxidative stress in the cell organelles stimulate several unfavourable effects in our body leading to various ailments, particularly ageing, hypertension, inflammatory reactions, diabetes, cancer, etc., that were found to depend on the accumulation of reactive oxygen species (ROS) (Lushchak 2011). Previous studies reported that the antioxidative agents were able to suppress the pro-inflammatory cyclooxygenase-2 (COX-2) and 5-lipoxygenase (5-LOX) enzymes that were responsible for inducing inflammatory responses through the release of inflammatory prostaglandins (PGE_2_ and PGF_2_α) and leukotrienes (LTB_4_) (Mitchell et al. 1994; D’Orazio et al. 2012). Thus, there is increased interest in pharmacological agents that can control or quench the free radicals from accumulating in the biological systems. The existing synthetic pharmacophores and nonsteroidal anti-inflammatory drugs (NSAIDs), which were reported to inhibit the free radical intermediates and pro-inflammatory mediators were recommended for limited usage due to their adverse effects (Schnitzer et al. 1999). The selective inhibition of inflammatory enzymes, such as COX-2/5-LOX, and oxidative stress inducing factors using naturally available pharmacological compounds can significantly lessen the adverse effects of the synthetic NSAIDs, corticosteroids and painkillers.

The natural products from marine organisms, such as molluscs or bivalves, which are adapted to the adverse living conditions in the oceanic ecosystem, have been considered as potential antioxidants. These organisms were reported to biosynthesize bioactive secondary metabolites as an adaptive mechanism, and these were recognized as valuable pharmacophores for use against various oxidative stress and inflammatory disorders (Gonzalez et al. 2015; Chakraborty et al. 2016). The bioactive properties of bivalves, mainly anti-inflammatory, antioxidant, antitumour properties etc. were reported in the previous literatures (Benkendorff 2010; Nagash et al. 2010; Chakraborty et al. 2016). The secondary metabolites from marine organisms belonged to different classes, such as heterocyclics, terpenes or steroids, and their activities were found to be closely related with their chemical structures. Isopimarane and pimarane metabolites were classified as significantly important class of diterpenoids with interesting pharmacological properties, such as antidiabetic, antioxidant, anti-HIV and antimicrobial activities, and were reported in marine organisms (Porto et al. 2009; Sun et al. 2012; Xia et al. 2015). Although rare in occurrence, three cytotoxic isopimarane diterpenoids from *Excoecaria acerifolia* Didr. (Euphorbiaceae) (Huang et al. 2013) and brominated pimaranes from marine algae, *Laurencia obtusa* (Hudson) Lamouroux (Rhodomelaceae) (Takeda et al. 1990) were reported in previous literature. An *ent*-pimarane diterpenoid tedanol was isolated from the marine sponge *Tedania ignis* (Duchassaing & Michelotti, (Tedaniidae), and was reported to possess potential anti-inflammatory activity against pro-inflammatory COX-2 enzyme (Costantino et al. 2009). The bioactive diterpenoids with pimarane skeletons were also described from the marine molluscs (sea hares), *Aplysia dactylomela* Rang, (Aplysiidae) (Schmitz et al. 1982) and *Aplysia pulmonica* Gould, (Aplysiidae) (Bian et al. 2014). The commercially available anti-inflammatory lipid extract of New Zealand green-lipped mussel *Perna canaliculus* Gmelin, (Mytilidae) known as Lyprinol^®^ and anti-inflammatory supplement Cadalmin^TM^ Green Mussel extract (Cadalmin^TM^ GMe) from Asian green mussel *Perna viridis* Linn. (Mytilidae) are prominent examples of pharmacologically effective agents from bivalve molluscs (Whitehouse et al. 1997; Chakraborty et al. 2014).

The bivalve clam *P. malabarica* considered in the present study are predominantly available seafood resource distributed in the coastal waters of southwestern India. Previous studies at our laboratory reported this species as a valuable source of balanced nutritional elements, such as C_20–22_*n*-3 polyunsaturated fatty acids, essential amino acids and minerals (Joy & Chakraborty 2016). Antioxidant, anti-inflammatory, antidiabetic and antihypertension potentials of the crude solvent extracts of *P. malabarica* were documented in our earlier study (Joy et al. 2016). As a sequel of our previous studies, it is of interest to isolate and characterize the bioactive pharmacophores that are responsible to impart potential antioxidative and anti-inflammatory properties. Based on this background, the present paper revealed the isolation and characterization of a new rearranged isopimarane norditerpenoid derivative from yellow-foot bivalve clam, *P. malabarica*, based on comprehensive spectroscopic analyses including mass and two-dimensional nuclear magnetic resonance spectroscopic experiments (2D NMR). In connection with that, the antioxidant [1,1-diphenyl-2-picrylhydrazyl (DPPH) and 2,2′-azino-bis-3-ethylbenzothiozoline-6-sulfonic acid diammonium salt (ABTS^+^)] and anti-inflammatory (COX-1, COX-2 and 5-LOX) properties of this C-19 norditerpene were carried out to establish its pharmacological potential.

## Materials and methods

### General experiments

The reagents and solvents were procured from E-Merck (Darmstadt, Germany), and were of spectroscopic/chromatographic/analytical grades. Fourier-transform infrared spectrum (FTIR) (on KBr) was recorded in a Perkin-Elmer Series 2000 FTIR spectrophotometer with scanning between 4000 and 400 cm^−1^. 1D (500 MHz for ^1^H, 125 MHz for ^13^C, Distortionless enhancement by polarization transfer, DEPT) and 2D (^1^H-^1^H COSY, correlation spectroscopy; HSQC, heteronuclear single-quantum correlation spectroscopy; HMBC, heteronuclear multiple-bond correlation spectroscopy; and NOESY, nuclear overhauser effect spectroscopy) NMR spectra were recorded on a Bruker AVANCE III 500 MHz (AV 500) spectrometer (Bruker, Karlsruhe, Germany) in CDCl_3_ as aprotic solvent at ambient temperature with TMS as internal standard (*δ* 0 ppm). Gas chromatography-mass spectrometric (GC-MS) analyses were performed in electronic impact (EI) ionization mode in a Perkin-Elmer Clarus 680 GC-MS fitted with a Elite 5 MS nonpolar, bonded phase capillary column (50 m × 0.22 mm i.d. × 0.25 μm film thicknesses). UV spectrum and *in vitro* spectroscopic assays were obtained on UV-Vis spectrophotometer (50 Varian Cary, Walnut Creek, CA). Analytical HPLC experiments were performed with a SPD M20A DAD (diode array detector, Kyoto, Japan) connected to a LC-20AD pump and C_18_ reverse phase column (Luna 250 x 4.6 mm, 5 μm, Phenomenex, Torrance, CA). The samples were freeze-dried by the lyophilization technique using Martin Christ alpha 1-4 LD Plus freeze-drier (Martin Christ, Osterode, Germany) and the concentration of solvent extracts was carried out by using a rotary vacuum evaporator (Heidolf, Schwabach, Germany). The chromatographic purification was carried out by flash chromatography (Biotage AB SP1-B1A, Sweden) on a silica gel (230–400 mesh, 12 g). Thin-layer chromatography (TLC) and preparatory TLC (PTLC) were carried out on pre-coated silica gel plates (Merck, Kieselgel-60F_254_).

### Sample collection and preparation of crude extracts

The clam samples, *P. malabarica* (10 kg) were freshly collected from Ashtamudi Lake (8°59′ N and 76°36′ E) situated along the southwest coast of India and a voucher with specimen number ICAR/CRP-HF/AC 368 was deposited in the repository of the Indian Council of Agricultural Research Consortium Research Platform on Health Food. The edible portion of the test material (6 kg) was separated from the shells before being homogenized and freeze dried by lyophilization. The dried powder (1.20 kg, yield 20.0%) was extracted with equal proportion of ethyl acetate (EtOAc) and methanol (MeOH) (1:1, v/v, 500 mL ×3) solvents at 40 °C and the extracts were filtered over anhydrous sodium sulphate (Na_2_SO_4_, 100 g), before being evaporated *in vacuo* by using a rotary evaporator (50 °C) to get a dark brown viscous residue (55.0 g, yield on dry basis 4.58%) of *P. malabarica* (Joy et al. 2016).

### Chromatographic purification of compound from *Paphia malabarica*

The crude extract of *P. malabarica* (45.0 g) was slurried with silica gel (4 g, 60–120 mesh), and packed into a column (1000 mm ×40 mm) containing silica (60–120 mesh). The column was initiated by eluting with 100% *n*-hexane followed by EtOAc and MeOH to obtain six pooled factions. The fraction PM_4_ (3.53 g, yield 7.84%) eluted at 70% EtOAc/*n*-hexane was flash chromatographed on a silica gel column (230–400 mesh) with a step gradient elution of *n*-hexane/EtOAc/MeOH at a collection wavelength of 258 nm to afford seven sub-fractions (PM_4-1_ to PM_4-7_) after TLC analyses. The fraction PM_4-4_, was found to possess greater antioxidant (DPPH and ABTS^+ ^scavenging) properties, and therefore, selected for further purification by preparative thin-layer chromatography (PTLC) over silica gel (GF_254_) using *n*-hexane/EtOAc (43:7, v/v) to afford the title compound (95 mg). Evaporation of solvents from the fractions followed by TLC over silica gel GF_254_ using EtOAc/*n*-hexane (15:85, v/v) supported its purity.

### Identification of isopimarane norditerpenoid derivative

Isopimarane norditerpenoid, 18 (4 → 14), 19 (4 → 8)-bis-abeo nor-isopimarane-1, 5-diene-3-yl-3β-methoxy propyl pentanoate: Amorphous white; m.p. 172–174 °C (decom.); UV (MeOH) *λ*_max_ (log ɛ): 270 nm (3.12); TLC (Si gel GF_254_ 15 mm; 15% EtOAc:*n*-hexane) *R*_f_: 0.65; R_t_ (GC): 23.32 min.; IR *ν*_max_ (KBr) cm^−1^: 2922.22, 2853.52 (C–Hν), 1722.04 (C = Oν), 1642.35 (C = Cν), 1375.15, 1260.54, 1035.15 (C–Oν); ^1^H (CDCl_3_, 500 MHz) *δ* 5.37 (1H, *J* = 7.24 Hz, dd), 5.38 (1H, *J* = 8.51 Hz, dd), 5.34 (1H, *J* = 6.61 Hz, t), 4.15 (2H, t), 3.64 (2H, t), 3.53 (1H, td), 2.81 (1H, dd), 2.33 (2H, t), 2.29 (2H, d), 2.01 (2H, d), 1.85 (1H, m), 1.83 (1H, t), 1.63 (2H, m), 1.61 (2H, m), 1.49 (1H, m), 1.44 (2H, m), 1.15 (2H, d), 1.12 (1H, m), 1.08 (1H, t), 1.01 (3H, s), 0.91 (1H, m), 0.89 (3H, t), 0.87 (3H, t), 0.86 (3H, d), 0.68 (3H, s); ^13^C NMR (125 MHz, CDCl_3_): 178.23, 140.72, 130.01, 129.71, 121.72, 71.83, 68.35, 65.03, 56.77, 50.14, 42.32, 42.24, 39.52, 37.25, 36.50, 33.87, 31.93, 29.70, 28.23, 24.75, 22.82, 22.69, 21.09, 19.39, 18.72, 14.11, 11.86. The ^1^H-^1^H COSY and HMBC spectral data are shown in Table 1. HRMS *m/e* calcd for C_27_H_44_O_3_ 416.3290, found 416.3295 [(M)^+^].

**Table 1.** NMR spectroscopic data in CDCl_3_.

**1**				
C. No.	^13^C	^1^H (int., mult., *J* in Hz)^a^	^1^H-^1^H COSY	HMBC
1	129.71	5.37 (1H, dd overlap, 7.24)	H-10	C-11
2	130.01	5.38 (1H, dd overlap, 8.51)	H-3	C-4, 21
3	71.83	3.53 (1H^α^, td)	H-4	–
4	42.24	2.29 (2H, d)	–	C-5, 6, 3
5	140.72	–	–	–
6	121.72	5.34 (1H, t, 6.61)	H-7	–
7	39.52	2.01 (2H, d)	–	C-6
8	42.32	–	–	–
9	50.14	0.91 (1H, m)	H-11	C-13, 14
10	28.23	2.81 (1H, dd)	–	C-14
11	31.93	1.49 (1H^β^, m), 1.85 (1H^α^, m)	H-12	–
12	37.25	1.83 (1H, t), 1.08 (1H, t)	–	–
13	36.50	–	–	–
14	56.77	1.12 (1H^β^, m)	H-18	–
15	22.69	1.15 (2H, d)	H-16	C-18, 19
16	14.11	0.89 (3H, t)	–	C-18, 19
17	11.86	0.68 (3H^α^, s)	–	C-12, 14, 8
18	22.82	0.86 (3H, d)	–	C-19, 11, 8
19	19.39	1.01 (3H^α^, s)	–	C-16, 13, 9, 5
20	68.35	3.64 (2H, t)	H-21	C-3
21	29.70	1.61 (2H, m)	H-22	–
22	65.03	4.15 (2H, t)	–	–
23	178.23	–	–	–
24	33.87	2.33 (2H, t)	H-25	C-25, 23
25	24.75	1.63(2H, m)	H-26	C-24, 23
26	21.09	1.44 (2H, m)	H-27	C-24, 25
27	18.72	0.87 (3H, t)	–	–

^1^H NMR spectra recorded using Bruker AVANCE III 500 MHz (AV 500) spectrometer (Bruker, Karlsruhe, Germany) in CDCl_3_ as aprotic solvent at ambient temperature with TMS as the internal standard (*δ* 0 ppm).

The ^1^H NMR spectra were recorded at 500 MHz, while the ^13^C NMR spectra were recorded at 125 MHz.

a Values in ppm, multiplicity and coupling constants (*J* = Hz) are indicated in parentheses.

The assignments were made with the aid of the ^1^H-^1^H COSY, HSQC, HMBC and NOESY experiments.

### Determination of antioxidant and anti-inflammatory activity

The antioxidant activities were evaluated using 1,1-diphenyl-2-picrylhydrazyl (DPPH) (Chew et al. 2008) and 2,2′-azino-bis-3-ethylbenzothiozoline-6-sulfonic acid diammonium salt (ABTS^+^) radical scavenging assays (Vijayabaskar & Shiyamala 2012). *In vitro* anti-inflammatory properties were determined by the percentage inhibition of pro-inflammatory cyclooxygenases (COX-2, COX-1) (Larsen et al. 1996) and 5-lipoxygenase (5-LOX) (Baylac & Racine 2003) enzymes. The free radical scavenging activities (DPPH and ABTS^+^) and enzyme inhibitory activities (COX-2, COX-1 and 5-LOX) of the tilted compound/standards with varying concentrations (0.25–2.00 mg/mL) were expressed as inhibition using the equation, inhibition (%) = {(absorbance of control − absorbance of sample/standards)/absorbance of control ×100}. The plots of inhibitory activities on radicals or enzymes were recorded and IC_50_ (concentration of samples at which it inhibits/scavenge 50% of enzyme/radical activities) values were calculated from the graph. The IC_50_ values (mg/mL) were determined from the linear regression curve of percentage inhibitions against the different concentrations of the compound or standards. The plot of scavenging or enzyme inhibitory activities were recorded and the IC_50_ (concentration of samples at which it inhibits/scavenge 50% of enzyme/radical activities) values were calculated from the graph. The structure-activity relationship analyses were carried out by calculating the hydrophobic descriptor (logarithm of octanol-water coefficient, log P_ow_), molar refractivity (MR) and polarizability (total polar surface area, tPSA) factors of the purified compounds using ChemDraw Ultra 8.0 database.

### Statistical analysis

One-way analysis of variance (ANOVA) was carried out with the Statistical Program for Social Sciences 13.0 (SPSS Inc., Chicago, IL, ver. 13.0) to assess significant differences between the means. The values were given as mean of triplicates ± standard deviation. The means of all triplicate parameters were examined for significance by ANOVA and the significant differences were represented as *p* < 0.05.

## Results and discussion

### Spectroscopic characterization of isopimarane norditerpenoid derivative (1)

**Figure 1.**
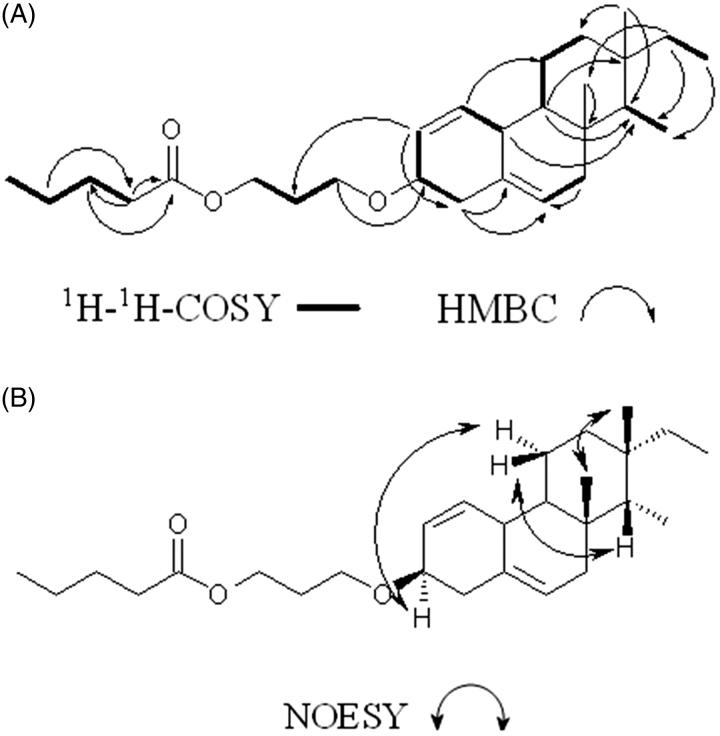
Key (A) ^1^H − ^1^H COSY, (B) HMBC and (C) NOESY correlations of 18 (4 → 14), 19 (4 → 8)-bis-abeo nor-isopimarane-1, 5-diene-3-yl-3β-methoxy propyl pentanoate.

The repeated column chromatographic separation of the crude ethyl acetate-methanol (EtOAc/MeOH) extract of *P. malabarica*, over silica gel, using mixtures of *n*-hexane/EtOAc/MeOH as mobile phase, yielded a C_19_ isopimarane norditerpenoid derivative (Table 1), as amorphous white powder. The title compound exhibited a molecular ion peak at *m/e* 416 (HRMS *m/e* found 416.3295 [M^+^]). The ^1^H and ^13^C NMR analyses confirmed the elemental composition as C_27_H_44_O_3_ having six degree of unsaturation related to two double bonds, three ring systems and a carboxylate group (Figures S1 and S2). The ^13^C NMR and DEPT data along with HSQC established the presence of 19 carbons including 4 methyls, 5 methylenes, 7 methines (in which one is oxygenated at *δ* 71.83) and 3 quaternary carbons, suggesting that the title compound was a norditerpene (Figures S2 and S3, S5). Notably, the *ent*-pimarane diterpenoid skeleton isolated from *Siegesbeckia orientalis* L. (Asteraceae) with 20 carbons including methyl (–CH_3_) group at C-10 was not apparent in the title compound, thus confirming the presence of norditerpenoid functionality (Wang et al. 2009). A downfield shift of *δ*H 3.53 attached to *δ*C 71.8 was due to the presence of oxygenated functionality at C-3. The characteristic quaternary carbon with greater chemical shift at *δ* 140.7 (C-5) was apparent as a result of adjacent vinylic group at *δ*H 5.34/*δ*C 121.7 (C-6) (Sun et al. 2012). The sequence of hydrogen and carbons were established with the help of HMBC and ^1^H-^1^H COSY correlations (Figures S4 and S6). The ^1^H-^1^H COSY spectrum exhibited six spin systems, which include H-1 (*δ* 5.37)/H-10 (*δ* 2.81); H-2 (*δ* 5.38)/H-3 (*δ* 3.53)/H-4 (*δ* 2.29); H-6 (*δ* 5.34)/H-7 (*δ* 2.01); H-9 (*δ* 0.91)/H-11 (*δ* 1.49, 1.85)/H-12 (*δ* 1.83, 1.08); H-14 (*δ* 1.12)/H-18 (*δ* 0.86); H-15 (*δ* 1.15)/H-16 (*δ* 0.89) (Figure 1(A)). The HMBC correlations from H-1 (*δ* 5.37) to C-11 (*δ* 31.93); H-2 (*δ* 5.38) to C-4 (*δ* 42.24); H-4 (*δ* 2.29) to C-5 (*δ* 140.2), C-6 (*δ* 121.7), C-3 (*δ* 71.8); H-7 (*δ* 2.01) to C-6 (*δ* 121.7) and H-9 (*δ* 0.91) to C-13 (*δ* 36.5), C-14 (*δ* 56.7) revealed the presence of tricyclic norditerpene framework (Figure 1(A)). The NOE correlations between *δ* 1.01 (H-19)/*δ* 0.68 (H-17) confirmed the relative configuration of the chiral centre as β and other correlation between *δ* 5.35 (H-6)/*δ* 1.99 (H-7), *δ* 3.49 (H-3), *δ* 2.31 (H-4) established that H-6 as well as H-3 were α configured (Figure S7). This stereo-chemical arrangement specifically at H-17 and H-15 (β and α, respectively) was comparable with the isolated isopimarane diterpenoids (Xia et al. 2015). The bulky –O-propyl pentanoate group appeared to be equatorially disposed, and therefore, the proton at the junction point (C-3) might be axial and α-oriented. The presence of NOEs between the axial methyl at C-14 and the methine proton H-3 belonging to the substituted rearranged isopimarane skeleton situated at the junction point connected with –O-propyl pentanoate was apparent (Figure 1(B)). The stereochemistry of oxygenated derivative at C-3 was further confirmed as β based on literature study of oxygenated pimarane (Sun et al. 2012) and isopimarane diterpenes (Huang et al. 2013). The usual *gem*-dimethyl group (C-18 and C-19) found at C-4 position in isopimarane and 20-nor-isopimarane diterpenoids were absent at C–l4 in the title compound (Wang et al. 2011). However, the -CH_3_ groups, such as C-18 and C-19 appeared at C-14 and C-8 positions, respectively, and therefore, it can be classified as 18 (4 → 14), 19 (4 → 8)-bis-abeo nor-isopimarane. The 3β-methoxy propyl pentanoate was recognized at C-3 by long-range coupling from H-2 (*δ* 5.38) to C-21 (*δ* 29.70) and H-20 (*δ* 3.64) to C-3 (*δ* 71.83). This linear chain having two spin systems from H-20 (*δ* 3.64)/H-21 (*δ* 1.61)/H-22 (*δ* 4.15) in the propyl moiety and H-24 (*δ* 2.33)/H-25 (*δ* 1.63)/H-26 (*δ* 1.44)/H-27 (*δ* 0.87) in the pentanoate chain. The occurrence of ester carbonyl carbon (*δ* 178.23) at C-23 of the pentanoate chain was verified by the HMBC long-range relations, such as H-24 (*δ* 2.33) to C-25 (*δ* 24.75), C-23 (*δ* 178.23); H-25 (*δ* 1.63) to C-24 (*δ* 33.87), C-23 (*δ* 178.23) and H-26 (*δ* 1.44) to C-24 (*δ* 33.87)/C-25 (*δ* 24.75). The distinctive IR stretching absorption bands at 2922.22 and 1722.04 cm^−1^ indicated C–H and C=O stretching vibrations, respectively, whereas those at 1375.15, 1260.54 cm^−1^ revealed the presence of C–O bending vibrations, thereby substantiated the structure of the title compound. Fragmentation of the molecular ion peak at *m/e* 416 [(M)^+^] was perceived to be accompanied by the loss of a C-4 fragment (butyl radical) resulting in an ion at *m/e* 359 (**1a**), and has been ascribed to 18(4 → 14), 19(4 → 8)-bis-abeo-nor-isopimarane-1,5-diene-3-yl-3β-methoxy formate (Figure S8). The fragment ion at *m/e* 286 (**1c**) undergo fragmentation to obtain fragment peak at *m/e* 256 (**1d**, 18(4 → 14), 19 (4 → 8)-bis-abeo-nor-isopimarane-1, 5-diene), which on subsequent rearrangement yielded the fragments with *m/e* 229 (**1e**), 206 (**1f**) and 185 (**1 g**) (Figure S8) that was comparable with earlier reports (Bromann et al. 2014). Based on all these spectral analyses, the purified compound was identified as C_19_ isopimarane norditerpenoid, 18 (4 → 14), 19 (4 → 8)-bis-abeo nor-isopimarane-1, 5-diene-3-yl-3β-methoxy propyl pentanoate.

The titled C_19_ isopimarane norditerpenoid exhibited no significant difference in scavenging DPPH and ABTS^+ ^free radicals (IC_50_ 0.65 and 0.78 mg/mL, respectively) compared to those displayed by the commercially available antioxidant, α-tocopherol (0.63 and 0.73 mg/mL, respectively) (*p* > 0.05). The anti-inflammatory effect (against 5-LOX enzyme) was significantly greater for the title compound (IC_50_ 0.75 mg/mL) compared to synthetic NSAID ibuprofen (IC_50_ 0.93 mg/mL; *p* < 0.05) (Table 2). It is of note that the NSAIDs are used for moderating the pathogenesis due to inflammatory pain and arthritis (Quan et al. 2008), although these drugs were reported to cause deleterious side effects, such as gastric ulcers, CVD and toxicosis on the various organs (Schnitzer et al. 1999). Notably, the adverse implications of NSAIDs were reported to be due to greater anti-COX-1 properties. COX-1 is a constitutive enzyme of gastrointestinal mucosa, a greater inhibition of this enzyme was found to be associated with gastro-intestinal ulcers in susceptible individuals. On the other hand, COX-2 and 5-LOX were reported to be the inducive pro-inflammatory enzymes, and their expression was found to be upregulated in response to inflammatory stimuli. Apparently, simultaneous inhibition of COX-2 and 5-LOX is vital to arrest the inflammatory response in affected individuals. A greater selectivity index (anti-COX-1_IC50_/anti-COX-2_IC50_) of the pharmacophores also signifies their greater selectivity and safety profile. Apparently, the greater selectivity index of the title compound (0.85) (Table 2) explained its lesser side effect than the nonsteroidal anti-inflammatory drug-based therapies (e.g., selectivity index of ibuprofen 0.44) (Botting 2006).

**Table 2.** Antioxidant and anti-inflammatory activities of the title compound from *P. malabarica* and the commercially available antioxidants and anti-inflammatory agents (*α*-tocopherol and ibuprofen).

	IC_50_ (mg/mL)	
Antioxidant activities	Isopimarane norditerpenoid	*α*-Tocopherol
^c^DPPH scavenging	0.65 ± 0.02^a^	0.63 ± 0.04^a^
^c^ABTS^+^ scavenging	0.78 ± 0.06^a^	0.73 ± 0.05^a^
Anti-inflammatory activities	Isopimarane norditerpenoid	Ibuprofen
^c^COX-1 inhibition	0.70 ± 0.02^a^	0.04 ± 0.00^b^
^c^COX-2 inhibition	0.82 ± 0.05^a^	0.09 ± 0.02^b^
^d^Selectivity index	0.85 ± 0.04^a^	0.44 ± 0.03^b^
^c^5-LOX inhibition	0.75 ± 0.06^a^	0.93 ± 0.11^b^

c The bioactivities were expressed as IC_50_ values (mg/mL).

The samples were analyzed in triplicate (*n* = 3) and expressed as mean ± standard deviation. Means followed by the superscript letters (a and b) within the same row indicate significant differences (*p* < 0.05).

d Selectivity index has been calculated as the ratio of anti-COX-1(IC_50_) and anti-COX-2 (IC_50_).

The antioxidative and anti-inflammatory activities of the isolated norditerpenoid from the marine bivalve clam, *P. malabarica* were correlated with the lipophilic/hydrophobic (log P_ow_), steric effect (MR) and polarizability (tPSA) factors that explained its structure-activity relations and drug-target interactions (Ajay et al. 1998). The hydrophobicity value (log P_ow_) for the titled C_19_ isopimarane norditerpenoid and α-tocopherol were calculated as 6.30 and 9.98, respectively, which was the ratio of 1-octanol to water partition coefficient. The antioxidant activity of title compound and α-tocopherol were comparable, even though the acceptable lipophilic levels of the former demonstrated its convenience and utility as a safer pharmacophore. Also, the steric descriptor for α-tocopherol (139.21) and isopimarane norditerpenoid (125.96) explained the relatively lesser bulk hindrance of the latter. The olefinic centres in the isopimarane along with the oxygenated and carboxylated side chain attached to the skeleton at C-3 enhanced its electronic properties and these electronegative functional moieties appeared to influence its antioxidative and anti-inflammatory activities. The commercially available NSAIDs, such as ibuprofen, were reported to inhibit both pro-inflammatory COX-1 and COX-2. The lesser selectivity ratio (anti-COX-1_IC50_/anti-COX-2_IC50_) of the NSAIDs also explained the larger selective inhibition of constitutive COX-1 that can cause severe side effects such as gastric-related health problems (Laneuville et al. 1994). Therefore, the search for compounds with specificity towards anti-COX-2 was preferred due to the lesser gastrointestinal difficulties as well as safer therapeutic profiles (Sprangler 1996). The naturally available norditerpenoid derivative reported in the present study was found to possess specific inhibition towards COX-2 activity than COX-1, and therefore, can be suggested as better anti-inflammatory lead molecule.

Previous reports of isopimarane and their derivatives from the natural resources envisaged their bioactive potential and pharmacological effects. The potential antioxidative property of *ent-*pimara-8(14), 15-diene from fungus was demonstrated by the DPPH radical scavenging activity (Bromann et al. 2014). The antibacterial potentials of rare pimarane derivatives with cyclopropane rings at C-3 and C-4 from the isolates of *A. pulmonica* were reported (Bian et al. 2014). The titled C_19_ isopimarane norditerpenoid enclosed with a straight chain of 3*-*methoxy propyl pentanoate at the C-3 position of isopimarane skeleton, thus accounted for its potential activity. The *ent*-pimarane derivative isolated from the sponge (*T. ignis*) exhibited strong *in vivo* inhibitory properties towards COX-2 and inflammatory cytokine-inducible nitric oxide synthase (iNOS) expression (Costantino et al. 2009). The pimarane diterpenoid, libertellenones, as a potent anticancer agent (Oh et al. 2005) and a C-19 diterpenoid pimarane from *Ephemerantha fimbriata* (Blume) P.F. Hunt & Summerh. (Orchidaceae) (Ma et al. 1998) were studied. The isolated isopimarane norditerpenoid can be a potential lead pharmacophore for therapeutic investigations. Also, the synthetic derivatives of particular compound with bioactive and safer functional groups with suitable physical properties and fewer side effects can be developed in future medications.

## Conclusions

To the best of our knowledge, 18 (4 → 14), 19 (4 → 8)-bis-abeo nor-isopimarane-1, 5-diene-3-yl-3β-methoxy propyl pentanoate represent the first description of C_19_ isopimarane norditerpenoid possessing the bis-abeo C_19_ norditerpenoid framework from a natural bivalve source. These unprecedented isopimarane derivative isolated from *P. malabrica* would be a potential natural alternative to the commercially available synthetic antioxidants and anti-inflammatory agents.

## References

[CIT0001] Ajay Walters WP, Murcko MA. 1998 Can we learn to distinguish between “drug-like” and “nondrug-like” molecules? J Med Chem. 41:3314–3324. 9719583 10.1021/jm970666c

[CIT0002] Baylac S, Racine P. 2003 Inhibition of 5-lipoxygenase by essential oils and other natural fragment extracts. Int J Aromather. 13:138–142.

[CIT0003] Benkendorff K. 2010 Molluscan biological and chemical diversity: secondary metabolites and medicinal resources produced by marine molluscs. Biol Rev Camb Philos Soc. 85:757–775. 20105155 10.1111/j.1469-185X.2010.00124.x

[CIT0004] Bian W-T, You Z-J, Wang C-Y, Shao C-L. 2014 Brominated pimarane diterpenoids from the sea hare *Aplysia pulmonica* from the South China Sea. Chem Nat Comp. 50:557–559.

[CIT0005] Botting RM. 2006 Inhibitors of cyclooxygenases: mechanisms, selectivity and uses. J Physiol Pharmacol. 57:113–124. 17218763

[CIT0006] Bromann K, Viljanen K, Moreira VM, Yli-Kauhaluom J. 2014 Isolation and purification of *ent*-pimara-8(14), 15-diene from engineered *Aspergillus nidulans* by accelerated solvent extraction combined with HPLC. Anal Methods. 6:1227–1234.

[CIT0007] Chakraborty K, Chakkalakal SJ, Joseph D, Asokan PK, Vijayan KK. 2016 Nutritional and antioxidative attributes of green mussel (*Perna viridis* L.) from the southwestern coast of India. J Aquat Food Prod Technol. 25:968–985.

[CIT0008] Chakraborty K, Chakkalakal SJ, Joseph D. 2014 Response of pro-inflammatory prostaglandin contents in anti-inflammatory supplements from green mussel *Perna viridis* L. in a time-dependent accelerated shelf-life study. J Funct Food. 7:527–540.

[CIT0009] Chew YL, Lim YY, Omar M, Khoo KS. 2008 Antioxidant activity of three edible seaweeds from two areas in South East Asia. LWT-Food Sci Technol. 41:1067–1072.

[CIT0010] Costantino V, Fattorusso E, Mangoni A, Perinu C, Cirino G, De Gruttola L, Roviezzo F. 2009 Tedanol: a potent anti-inflammatory *ent*-pimarane diterpene from the Caribbean Sponge *Tedania ignis*. Bioorg Med Chem. 17:7542–7547. 19800802 10.1016/j.bmc.2009.09.010

[CIT0011] D’Orazio N, Gammone AM, Gemello E, De Girolamo M, Cusenza S, Riccioni G. 2012 Marine bioactives: pharmacological properties and potential applications against inflammatory diseases. Mar Drugs. 10:812–833. 22690145 10.3390/md10040812 PMC3366677

[CIT0012] Oh D-C, Jensen PR, Kauffman CA, Fenical W. 2005 Libertellenones A–D: induction of cytotoxic diterpenoid biosynthesis by marine microbial competition. Bioorg Med Chem. 13:5267–5273. 15993608 10.1016/j.bmc.2005.05.068

[CIT0013] Gonzalez PM, Malanga G, Puntarulo S. 2015 Cellular oxidant antioxidant network: update on the environmental effects over marine organisms. Open Mar Biol J. 9:1–13.

[CIT0014] Huang SZ, Ma QY, Fang WW, Xu FQ, Peng H, Dai HF, Zhou J, Zhao YX. 2013 Three new isopimarane diterpenoids from *Excoecaria acerifolia*. J Asian Nat Prod Res. 15:750–755. 23777356 10.1080/10286020.2013.800862

[CIT0015] Joy M, Chakraborty K, Pananghat V. 2016 Comparative bioactive properties of bivalve clams against different disease molecular targets. J Food Biochem. 40:593–602.

[CIT0016] Joy M, Chakraborty K. 2016 Nutritional qualities of the low value bivalve mollusks *Paphia malabarica* and *Villorita cyprinoids* at the estuarine waters of southwestern coast of India. J Aquat Food Prod T. Online publication date 2016/2/1 (in press). doi: 10.1080/10498850.2015.1092486.

[CIT0017] Laneuville O, Breuer DK, DeWitt DL, Hla T, Funck CD, Smith WL. 1994 Differential inhibition of human prostaglandin endoperoxide H synthases-1 and -2 by nonsteroidal anti-inflammatory drugs. J Pharmacol Exp Ther. 271:927–934. 7965814

[CIT0018] Larsen LN, Dahl E, Bremer J. 1996 Peroxidative oxidation of leuco-dichloroluorescein by prostaglandin-H synthase in prostaglandin biosynthesis from polyunsaturated fatty acids. Biochim Biophys Acta. 1299:47–53. 8555252 10.1016/0005-2760(95)00188-3

[CIT0019] Lushchak V. 2011 Environmentally induced oxidative stress in aquatic animals. Aquat Toxicol. 101:13–30. 21074869 10.1016/j.aquatox.2010.10.006

[CIT0020] Ma GX, Yin L, Wang TS, Pan Y, Guo LW. 1998 A 19-carbon pimarane-type diterpenoid from *Ephemerantha fimbriata*. Pharm Biol. 36:66–68.

[CIT0021] Mitchell JA, Akarasereenont P, Thiemermann C, Flower RJ, Vane JR. 1994 Selectivity of nonsteroidal antiinflammatory drugs as inhibitors of constitutive and inducible cyclooxygenase. Proc Natl Acad Sci USA. 90:11693–11697. 10.1073/pnas.90.24.11693 PMC48050 8265610

[CIT0022] Nagash YS, Nazeer RA, Kumar NSS. 2010 *In vitro* antioxidant activity of solvent extracts of mollusks (*Loligo duvauceli* and *Donax strateus*) from India. World J Fish Mar Sci. 2:240–245.

[CIT0023] Porto TS, Rangel R, Furtado NAJC, de Carvalho TC, Martins CG, Veneziani RCS, Da Costa FB, Vinholis AHC, Cunha WR, Heleno VCG, et al 2009 Pimarane-type diterpenes: antimicrobial activity against oral pathogens. Molecules. 14:191–199. 19127247 10.3390/molecules14010191 PMC6253883

[CIT0024] Quan L-D, Thiele GM, Tian J, Wang D. 2008 The development of novel therapies for rheumatoid arthritis. Expert Opin Ther Pat. 18:723–738. 19578469 10.1517/13543776.18.7.723 PMC2491719

[CIT0025] Schmitz FJ, Michaud DP, Schmidt PC. 1982 Marine natural products: Parguerol, deoxyparguerol, and isoparguerol. New brominated diterpenes with modified pimarane skeletons from the sea hare *Aplysia dactylomela*. J Am Chem Soc. 104:6415–6423.

[CIT0026] Schnitzer J, Kamin M, Olson WH. 1999 Tramadol allows reduction of naproxen dose among patients with naproxen-responsive osteoarthritis pain. Arthritis Rheum. 42:1370–1377. 10403264 10.1002/1529-0131(199907)42:7<1370::AID-ANR10>3.0.CO;2-T

[CIT0027] Sprangler RS. 1996 COX-2 activity can reduce the level of toxicity for a given NSAID but may not be sufficient to overcome toxicities resulting from other mechanisms. Semin Arthritis Rheum. 26:436–447.

[CIT0028] Sun L, Li D, Tao M, Chen Y, Dan F, Zhang W. 2012 Scopararanes C-G: new oxygenated pimarane diterpenes from the marine sediment-derived fungus *Eutypella scoparia* FS26. Mar Drugs. 10:539–550. 22611352 10.3390/md10030539 PMC3347013

[CIT0029] Takeda S, Kurosawa E, Komiyama K, Suzuki T. 1990 The structures of cytotoxic diterpenes containing bromine from the marine red alga *Laurencia obtusa* (Hudson) Lamouroux. Bull Chem Soc Jpn. 63:3066–3072.

[CIT0030] Vijayabaskar P, Shiyamala V. 2012 Antioxidant properties of seaweed polyphenol from *Turbinaria ornata* (Turner) J. Agardh, 1848. Asian Pac J Trop Biomed. 2:S90–S98.

[CIT0031] Wang F, Cheng XL, Li Y-J, Shi S, Liu J-K. 2009 *ent*-Pimarane diterpenoids from *Siegesbeckia orientalis* and structure revision of a related compound. J Nat Prod. 72:2005–2008. 19813758 10.1021/np900449r

[CIT0032] Wang XN, Bashyal BP, Wijeratne EMK, U’Ren JM, Liu MX, Gunatilaka MK, Arnold AE, Gunatilaka AAL. 2011 Smardaesidins A-G, isopimarane and 20-nor-isopimarane diterpenoids from Smardaea sp., a fungal endophyte of the moss *Ceratodon purpureus*. J Nat Prod. 74:2052–2061. 21999655 10.1021/np2000864 PMC3371368

[CIT0033] Whitehouse MW, Macrides TA, Kalafatis N, Betts WH, Haynes DR, Broadbent J. 1997 Anti-inflammatory activity of a lipid fraction (lyprinol) from the NZ green-lipped mussel . Inﬂammopharmacology. 5:237–246. 17638133 10.1007/s10787-997-0002-0

[CIT0034] Xia X, Qi J, Liu Y, Jia A, Zhang Y, Liu C, Gao C, She Z. 2015 Bioactive isopimarane diterpenes from the fungus, *Epicoccum* sp. HS-1, associated with *Apostichopus japonicus*. Mar Drugs. 13:1124–1132. 25738327 10.3390/md13031124 PMC4377976

